# Supplementary material for: Assessment of Dietary and Lifestyle Quality among the Romanian Population in the Post-Pandemic Period
Source: Healthcare (Basel). 2024 May 14;12(10):1006. doi: 10.3390/healthcare12101006 (PMC11121699; doi:10.3390/healthcare12101006)
Supplement: Supplementary file 1 [file healthcare-12-01006-s001.zip › healthcare-2973869-supplementary.pdf]

# Supplementary Materials

## Article

## Assessment of Dietary and Lifestyle Quality among the Romanian Population in the Post-Pandemic Period

*QUESTIONNAIRE regarding adherence to the healthy lifestyle and balanced diet of the Romanian population*

***Personal data:***

***1. Please mention your age (in years):***

***2. Please mention your gender:***

Male

Feminine

Other

***3. Please mention your current residence:***

Town

Commune/Village

***4. Please mention your employment status:***

Unemployed

Socially assisted

Householder

Retired

Student

Teleworking

I go to work every day

I work in a mixed regime (telework and commuting)

***5. Please state your marital status:***

Single

Divorced / separated

Married

***6. Please mention the Level of education:***

General/primary studies

Secondary education (baccalaureate degree)

Post-secondary studies

Higher education (bachelor's degree)

Postgraduate studies (master's, residency, doctorate, other specializations)

***Anthropometric data***

***7. Please mention your weight (in kg):***

***8. Please mention your height (in cm):***

***Eating habits:***

***9. What is the main type of dietary fat consumed?***

Margarine

Lard, tallow

Butter

Refined vegetable oil

Extra virgin or virgin vegetable oil

***10. How many servings of vegetables (approx. 100 g) do you eat every day?***

Very rarely or not at all
One
Two
Three
More than three

***11. How many servings of fruit (approx. 100 g) do you eat every day?***

Very rarely or not at all
One
Two
Three
More than three

***12. How often do you eat meat?***

Very rarely or not at all
Once a week
Twice a week
More than 2 times a week
Daily

***13. How often do you consume carbonated or sweetened drinks (1 portion = 330 ml, a glass)?***

Daily
More than 2 times a week
Twice a week
Once a week
Very rarely or not at all

***14. How often do you consume alcoholic beverages (1 glass of wine=125ml, 1 glass of soft drink spirits =50ml)?***

Daily more than one serving
One serving daily
More than 2 times a week
Twice a week
Once a week
Very rarely or not at all

***15. How often do you eat fish or seafood?***

Very rarely or not at all
Once a week
Twice a week
More than 2 times a week
Daily

***16. How often do you eat sweets / pastries?***

Daily
More than 2 times a week
Twice a week
Once a week
Very rarely or not at all

***17. How often do you eat pasta, rice or other grains?***

Very rarely or not at all
Once a week
Twice a week
More than 2 times a week
Daily

***18. How much bread do you eat per day?***

More than 12 slices

8-12 slices
5-7 slices
1-4 slices
Very rarely or not at all

***19. How often do you consume dairy products?***

Very rarely or not at all
Once a week
Twice a week
More than 2 times a week
Daily

***20. How many eggs do you eat per week?***

Very rarely or not at all
1 - 2 eggs
3 - 4 eggs
5 - 7 eggs
More than 7 eggs

***21. Which food category do you eat most often?***

Fast food products
Pizza, snacks, pastries, sweets
Products made from sausages and preserves
Food cooked in restaurants
Home cooked food

***22. What type of cooked food do you eat most often?***

Fried foods
Food prepared by cooking on wood or coals
Grilled food
Food prepared in the oven
Boiled or steamed foods
Non-thermally processed foods

***23. How much water do you drink per day?***

Under 1L
1 L
2 L
3 L
Over 3 L

***24. Which category of liquids do you tend to consume most often?***

Alcoholic drinks: sparkling drinks/wine, beer, etc.
Carbonated or sweetened non-alcoholic drinks
Coffee
What do you have
Plain water and natural juices

***25. Which category of food products predominates in the daily diet?***

Produce
Fish and seafood preparations
Cereals and pasta
Dairy products
Meat and meat preparations
Sweets and pastries
Foods high in fat

***26. What type of meat do you eat most often?***

Fish and/or seafood
Bird meat
Rabbit meat
Beef

Game meat
Pork
Mutton
Goat meat
Other
I don't eat meat

***Lifestyle:***
***27. How are meals distributed per day?***
Consuming 1-2 meals a day without a fixed schedule
I eat 3 meals a day without a fixed schedule
I eat 3 meals a day and 1-2 snacks without a fixed schedule
Consume 3 meals a day according to a fixed schedule
Consume 3 meals a day and 1-2 snacks according to a fixed schedule
***28. How do you rate the amount of food consumed daily?***
I consider myself a chaotic, excessive eater
I believe that I eat chaotically, insufficiently
Weighted food consumption, without excesses
Consume food according to the needs of the body by monitoring the weight
I eat food according to the ration established by a specialist
***29. Do you think that your diet has affected your health?***
I do not know
Yes, because I eat junk food
Yes, because I overeat
Yes, because I don't eat enough
No
***30. When you serve the meal how do you do it?***
I generally eat in a hurry
During the meal I usually do something else
They serve the meal quietly and unhurriedly
*31. Do you think you need the advice of a nutritionist?*
Yes, to help me choose healthy foods
Yes, to help me eat weighted
No
***32. Which of the following factors do you consider to be most likely to affect your current state of health?***
Overwork
Sleep quality
The stress
Unhealthy diet
Lack of movement
pollution
Excessive smoking
Excessive consumption of alcohol or drugs
Financial problems
***33. What type of diet are you currently on?***
Normal, omnivorous diet
Vegetarian diet/variants
Vegan diet/variants
Ketogenic diet
Mediterranean diet
Other
***34. Have you ever gone on a diet to lose weight?***

Yes, often
Yes, very rarely
No

***35. What type of activity do you do?***

Work in difficult and dangerous conditions (construction site, factory, mine, etc.)
Work in front of the computer or special devices
Office work or activity with minimal movement
Work standing
Work outdoors in non-hazardous conditions

***36. Do you usually do sports / exercise?***

Not
Yes, very rarely
Yes, 2-3 times a week
Yes, daily under an hour
Yes, daily at least one hour

***37. Where do you do sports / exercise?***

The home
Outdoor
Gym
I don't do sports / exercise

***38. Do you smoke?***

Yes, excessively daily
Yes, daily 1-2 cigarettes
Yes, 2-3 times a week
Yes, occasionally
No

***39. How many hours a night you usually sleep?***

I have frequent insomnia
Under 7 hours per night
Over 9 hours a night
7-9 hours a night

***40. How do you rate the state of your immune system?***

I consider myself to have a strong immune system
I believe I have a weakened immune system
I regularly use various methods to strengthen the immune system

***41. What do you consider to be the main factors affecting your mental state?***

**Health problems**
Fatigue state
The stress
Overwork
Problems at work
Lack of communication, isolation
Inappropriate surroundings
Family problems
Financial problems

***42. What kind of problems do you encounter?***

I am often tired
I am often nervous
I am often depressed
I have frequent panic attacks / anxiety states
I eat emotionally
I'm not hungry

I'm fine, I have no problem

***43. Do you go to specialist doctors when you have health problems?***

Never
Yes, only in very serious cases
Yes, generally when self-medication doesn't work
Yes, when the state of health deteriorates more
Yes, every time there are problems

***44. Do you regularly assess your health?***

No
Yes, very rarely
Yes, after two years
Yes, once a year
Yes, at least twice a year

***45. How much time do you spend on average per day in front of the computer, tablet, phone or TV?***

Over 8 hours
6-7 hours
4-5 hours
2-3 hours
Under 1 hour

***46. What type of chronic conditions do you have?***

Arterial Hypertension
Cardiovascular diseases
Diabetes
Gout
Obesity
Pulmonary diseases
Renal diseases
Autoimmune diseases
Gastric conditions
Liver diseases
Respiratory diseases
Rheumatic diseases
Bone disorders
Mental disorders
Other
I am clinically healthy
I do not know

***47. What do you feel is currently lacking in a healthier lifestyle?***

Free time
Peaceful sleep, rest
Financial resources
Advice from a nutritionist
Access to quality medical services
Solving stressful problems
Communication and socialization
Reducing time spent on social networks or various sites
Nutritional knowledge
Movement, physical activity
Quality food products
Nothing

***48. How do you spend your free time?***

Watching movies and TV programs
On social networks or various websites

Reading
DIY (Do It Yourself)
With family or friends
Outdoor activities
Participation in cultural activities
Participation in charity events
Participation in training courses
Other ways.
